# Sustainability in health care by allocating resources effectively (SHARE) 4: exploring opportunities and methods for consumer engagement in resource allocation in a local healthcare setting

**DOI:** 10.1186/s12913-017-2212-5

**Published:** 2017-05-05

**Authors:** Claire Harris, Henry Ko, Cara Waller, Pamela Sloss, Pamela Williams

**Affiliations:** 1 School of Public Health and Preventive Medicine, Monash University, Clayton, VIC Australia; 2 Centre for Clinical Effectiveness, Monash Health, Clayton, VIC Australia; 3 NHMRC Clinical Trials Centre, Sydney Medical School, University of Sydney, Sydney, NSW Australia; 4 Consumer Representative, Monash Health, Clayton, VIC Australia

**Keywords:** Consumer, Community, Engagement, Disinvestment, De-adopt, Decommission, Health technology, TCP, Resource allocation, Decision-making

## Abstract

**Background:**

This is the fourth in a series of papers reporting a program of Sustainability in Health care by Allocating Resources Effectively (SHARE) in a local healthcare setting. Healthcare decision-makers have sought to improve the effectiveness and efficiency of services through removal or restriction of practices that are unsafe or of little benefit, often referred to as ‘disinvestment’. A systematic, integrated, evidence-based program for disinvestment was being established within a large Australian health service network. Consumer engagement was acknowledged as integral to this process. This paper reports the process of developing a model to integrate consumer views and preferences into an organisation-wide approach to resource allocation.

**Methods:**

A literature search was conducted and interviews and workshops were undertaken with health service consumers and staff. Findings were drafted into a model for consumer engagement in resource allocation which was workshopped and refined.

**Results:**

Although consumer engagement is increasingly becoming a requirement of publicly-funded health services and documented in standards and policies, participation in organisational decision-making is not widespread. Several consistent messages for consumer engagement in this context emerged from the literature and consumer responses. Opportunities, settings and activities for consumer engagement through communication, consultation and participation were identified within the resource allocation process. Sources of information regarding consumer values and perspectives in publications and locally-collected data, and methods to use them in health service decision-making, were identified. A model bringing these elements together was developed.

**Conclusion:**

The proposed model presents potential opportunities and activities for consumer engagement in the context of resource allocation.

**Electronic supplementary material:**

The online version of this article (doi:10.1186/s12913-017-2212-5) contains supplementary material, which is available to authorized users.

## About SHARE

*This is the fourth in a series of papers exploring a program of Sustainability in Health care by Allocating Resources Effectively (SHARE). The SHARE Program is an investigation of concepts, opportunities, methods and implications for evidence-based investment and disinvestment in health technologies and clinical practices in a local healthcare setting. The papers in this series are targeted at clinicians, managers, policy makers, health service researchers and implementation scientists working in this context. This paper reports the process of developing a model to integrate consumer views and preferences into an organisation-wide approach to resource allocation.*

## Background

Removal or restriction of health technologies and clinical practices (TCPs) that are unsafe or of little benefit, often referred to as ‘disinvestment’, has the dual advantage of improving patient outcomes and enabling more effective use of available resources.

Leaders at Monash Health (previously Southern Health), a large health service network in Melbourne, Australia, sought to explore possibilities for disinvestment within an organisation-wide, systematic, integrated, evidence-based approach to allocation of resources. The ‘Sustainability in Health care by Allocating Resources Effectively’ (SHARE) Program was undertaken by the Centre for Clinical Effectiveness (CCE), an in-house resource to facilitate Evidence Based Practice. An overview of the SHARE Program, a guide to the SHARE publications and further details about Monash Health and CCE are provided in the first paper in this series [1].

**Fig. 1:**
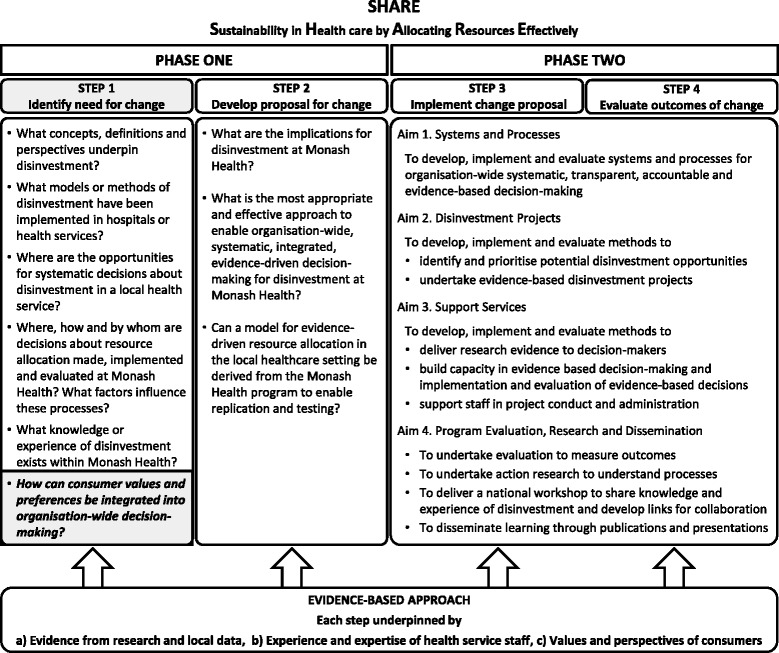
Overview of SHARE Program

Although the disinvestment literature has broadened considerably over the past two decades, there is little information to guide regional health authorities or local facilities in how they might take a systematic organisation-wide approach to disinvestment [2–11]. In the absence of guidance from the literature, a two-phased process was proposed to identify and then evaluate potential opportunities for disinvestment at Monash Health (Fig. 1). The aim of Phase One was to understand concepts and practices related to disinvestment and the implications for a local health service and, based on this information, to identify potential settings and methods for decision-making. The aim of Phase Two was to implement and evaluate the proposed methods to determine which were sustainable, effective and appropriate at Monash Health. The findings are reported in this thematic series [1, 12–21].

One of the aims of the SHARE Program was to explore ways to identify, capture and incorporate consumer perspectives into resource allocation decisions. The importance of public participation at all levels of the health system is widely recognised and has been described as a right and responsibility of healthcare consumers [22–24]. The primary goal of public involvement in health policy and service delivery is to ensure that decisions reflect the needs, concerns, values, culture, ideas and attitudes of consumers for whom the system exists and citizens who provide the resources for the system [23, 25–27]. Community and consumer participation is increasingly a requirement of publicly-funded health services. In Australia this is reflected in national standards and state policies [24, 28].

Monash Health was committed to consumer involvement in decision-making for establishment and delivery of the program. However, unlike decision-making contexts which are linked to a particular condition, service, population or other specified group, the SHARE Program was an organisation-wide approach that affected all patients across a multi-campus health service. This presented a challenge to the organisation in identifying and engaging consumer representatives to participate in these generic systems and processes and capturing consumer views and preferences to inform the activities.

**Fig. 2:**
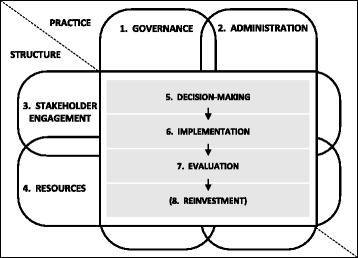
Conceptual framework of relationships between components of organisational infrastructure for resource allocation (from Harris et al [13] with permission)

Another SHARE project identified that, while decision-making is a key component of resource allocation, there are seven additional components required for achievement of this task [13]. The eight components are Governance, Administration, Stakeholder engagement, Resources, Decision-making, Implementation, Evaluation and, in some situations, Reinvestment. A framework illustrating the relationships between components demonstrates that stakeholder engagement should be integrated within the structure and practice of all the other components (Fig. 2, Table 1) [13]. Consumers are clearly stakeholders in allocation of health service resources and potentially have contributions to make in each of the eight components.

**Table 1:** Structure and practice elements of components of organisational infrastructure for resource allocation (from Harris et al [13] with permission)

Components	Structure (Who, What)	Practice (How)
1. Governance	▪ Overseers ▪ Policies for decision-making ▪ Transparency and accountability in all structures ▪ Requirements for addressing conflict of interest^a^ ▪ Requirements for monitoring, evaluation and improvement of systems and processes ▪ Requirements for reporting	▪ Oversight ▪ Procedures, guidelines, protocols for decision-making ▪ Transparency and accountability in all practices ▪ Methods of addressing conflict of interest ▪ Methods of monitoring, evaluation and improvement of systems and processes ▪ Methods of reporting
2. Administration	▪ Administrators ▪ Requirements for administration ▪ Relationships and coordination ▪ Communication	▪ Methods of administration, coordination, communication and collaboration
3. Stakeholder engagement	▪ Clinicians, Managers, Consumers, Technical experts, Funders, other relevant parties ▪ Requirements for stakeholder engagement	▪ Methods of identification, recruitment and engagement
4. Resources	▪ Funding sources ▪ Allocation of staff ▪ Access to experts or ways to gain expertise ▪ Information sources ▪ Requirements for resources	▪ Provision of appropriate and adequate funding, time, skills/training, information ▪ Utilisation of resources
5. Decision-making	▪ Decision-makers – Clinicians – Authorised individuals – Authorised groups ▪ Scope of decisions ▪ Type of decisions ▪ Requirements for decision-making	▪ Methods of decision-making – Identification of need/application – Decision criteria – Ascertainment and use of evidence – Reminders and prompts to consider disinvestment – Deliberative process – Documentation and dissemination
6. Implementation	▪ Purchasers ▪ Requirements for purchasing	▪ Methods of purchasing
	▪ Policy and guidance developers ▪ Requirements for policies and guidance documents	▪ Methods of policy and guidance development
	▪ Implementers ▪ Requirements for implementation	▪ Methods of project management ▪ Methods of change management
7. Evaluation	▪ Evaluators ▪ Requirements for evaluation ▪ Type and source of data collected	▪ Methods of evaluation
8. (Reinvestment)	Requirements for reinvestment/reallocation	Methods of reinvestment/reallocation

^a^Requirement is used in the sense of performance stipulated in accordance with policies, regulations, standards or similar rules/obligations

Although there is extensive literature on the effects of patient involvement in decisions about their own care [29–31], there is little evidence about the impact of public participation in decisions for healthcare policy and service delivery or the effectiveness of different types of engagement [25, 32–34]. There are numerous guides to public involvement in health service decision-making which provide information about potential engagement strategies, including a framework for patient involvement in decisions about use of health technologies at the local level [27], but there is no guidance on methods or frameworks for involving consumers in an organisation-wide approach for making, implementing and evaluating the whole range of resource allocation decisions [35]. The methods of public involvement in health policy decisions through Citizen Councils have been described at national [36] and state/provincial [37] levels and their characteristics summarised [26]. However Citizen Councils are independent from, and operate in parallel to, the institutional decision-making processes, which is in contrast to the Monash Health aim of integration within decision-making processes. There are many examples of participation of consumers alongside other stakeholders in project settings, in randomised controlled trials [32] and even in the context of disinvestment [9, 38–41], but these are limited to decision-making on a specified topic and do not address implementation or evaluation of the decision or the governance, administration and resources that underpin the processes. A systematic review on this topic [42] failed to find any frameworks or information for involving consumers in disinvestment decisions and these and other authors note an urgent need to develop methods to capture and utilise consumer perspectives in the context of resource allocation [10, 37, 42–44].

### Aims

The aims of this project were to document current practice and identify additional opportunities and methods to integrate consumer views and preferences into decision-making for resource allocation at Monash Health.

The aim of this paper is to incorporate the findings of this investigation into a model for consumer engagement and use of consumer evidence within an integrated, systematic, organisation-wide approach to resource allocation.

### Research question

How can consumer and community values and preferences be systematically integrated into organisation-wide decision-making for resource allocation?

## Methods

### Model for evidence-based change

The SHARE Program was undertaken using the SEAchange model for Sustainable, Effective and Appropriate change in health services [45]. The model involves four steps: identifying the need for change, developing a proposal to meet the need, implementing the proposal and evaluating the extent and impact of the change. Each step is underpinned by the principles of evidence-based practice to ensure that the best available evidence from research and local data, the experience and expertise of health service staff and the values and perspectives of consumers are taken into account. Steps 1 and 2 of the model map to Phase One of the SHARE Program and Steps 3 and 4 correspond to Phase Two. The research question for this paper is highlighted in Fig. 1.

### Literature review

A two-step systematic review protocol was developed (Additional file 1). The first step was to identify existing synthesised evidence and appraise it for quality and applicability; if no suitable publications were identified then a search of the primary research literature would follow. Relevant high-quality synthesised evidence in the form of guidance documents for consumer engagement were identified in the first step hence no further searches were undertaken. A second review limited to PubMed, The Cochrane Library and the Google internet search engine was conducted to ascertain more recent synthesised evidence prior to developing the model for this paper.

### Interviews and workshop

#### Participants

SHARE Consumer Working Group: three consumer representatives with experience in organisation-wide decision-making related to resource allocation as members of committees overseeing introduction of new TCPs and development of policies and procedures, and SHARE project team members

Monash Health Quality Manager and Consumer Engagement Manager: responsible for consumer-related activities

Monash Health Community Advisory Committee: a legislated advisory body to the health service Board providing consumer, carer and community perspectives

Monash Health staff who made organisation-wide decisions regarding resource allocation for TCPs

Monash Health project staff with experience in disinvestment-related activities

#### Data collection and analysis

Structured and semi-structured interviews and a structured workshop were undertaken. Details are provided in Additional file 1. A draft record of interview was sent to interviewees for clarification, comment and/or amendment as required. Responses were collated and analysed thematically by either content analysis [46] to identify emergent themes, or framework analysis [47] when categories had been specified *a priori*. Findings were presented in detailed reports used for project decision-making and planning. These reports have been synthesised to address the research question for this paper.

### Development of the model

#### Concepts

**Fig. 3:**
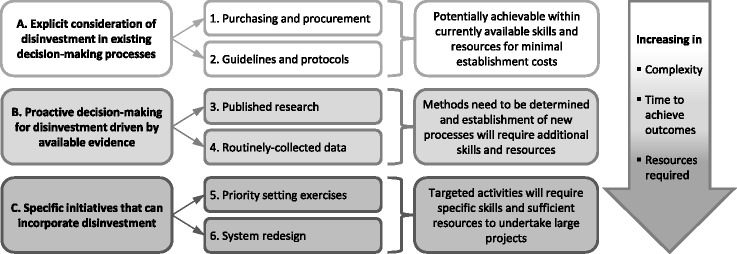
Conceptual framework of potential settings and methods for disinvestment decisions in health service systems and processes (from Harris et al [12] with permission)

**Fig. 4:**
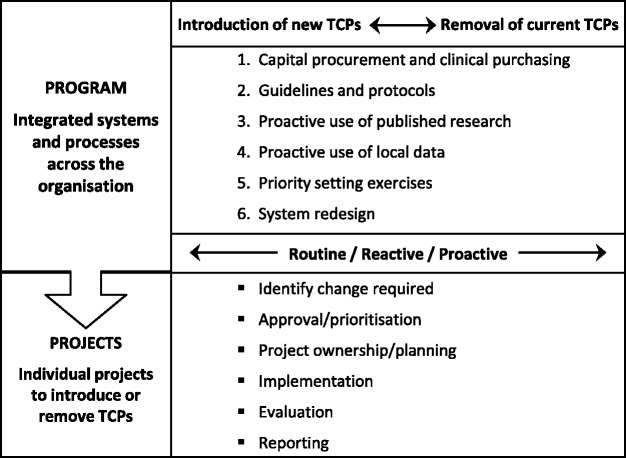
Conceptual framework of SHARE program (from Harris et al [15] with permission)

Concepts were identified from the literature, interviews and workshop, and three conceptual frameworks developed in other SHARE projects (Figs. 2, 3 and 4) [12, 13, 15]. Examples of activities within the key concepts were drawn from the literature and consumer feedback.

#### Relationships

Relationships were developed by aligning the concepts from the literature, interviews and workshop with the components of the resource allocation process (Fig. 2).

#### Definitions

Definitions were derived from the literature where possible. Two definitions were developed for the purposes of this model.

#### Drafting and refinement

A framework is made up of a set of concepts and the relationships between the concepts to facilitate the development of propositions. The purpose of a framework is to provide a frame of reference, organise and focus thinking and assist interpretation. Frameworks are descriptive, tend to be high-level and can apply to a wide variety of situations [48, 49]. An initial framework drafted to demonstrate potential consumer involvement in the six potential settings for disinvestment in a local healthcare setting was presented and discussed in the Community Advisory Committee workshop.

A model is more precise and more prescriptive than a framework. It is narrower in scope, the concepts are well defined and the relationships between them are specific. Models are representations of the real thing [48, 49]. The draft framework was revised based on the outcomes of the workshop and expanded to incorporate the additional elements from the other two frameworks (Figs. 3 and 4), concepts arising from the literature, and interview and workshop data. The level of detail describing and prescribing activities, methods and sources of information make this version a model.

#### Analysis and synthesis

The robustness and usefulness of the proposed model were analysed and synthesised using the domains and criteria outlined for this purpose by Rycroft-Malone and Bucknall [48]. The resulting summary enables potential users to identify models that meet their needs.

## Results and discussion

### How can consumer and community values and preferences be integrated into organisation-wide decision-making for resource allocation?

The literature review identified systematic reviews, frameworks, toolkits and guidance for consumer engagement [34, 50–59]. Of particular relevance to this project were documents developed for the Australian healthcare setting [51–53] and others focused on decisions about use of health technologies [57, 58].

Forty-seven staff members participated in the interviews and workshops. The responses provided details of current practice and the views of Monash Health consumers and staff concerning potential systems and processes.

Several key messages regarding an organisational approach to consumer engagement emerged from the literature. The same themes were also evident in the local responses. These messages are also consistent with more recently synthesised evidence and guidance for consumer engagement [27, 31, 33, 60–68].

**Fig. 5:**
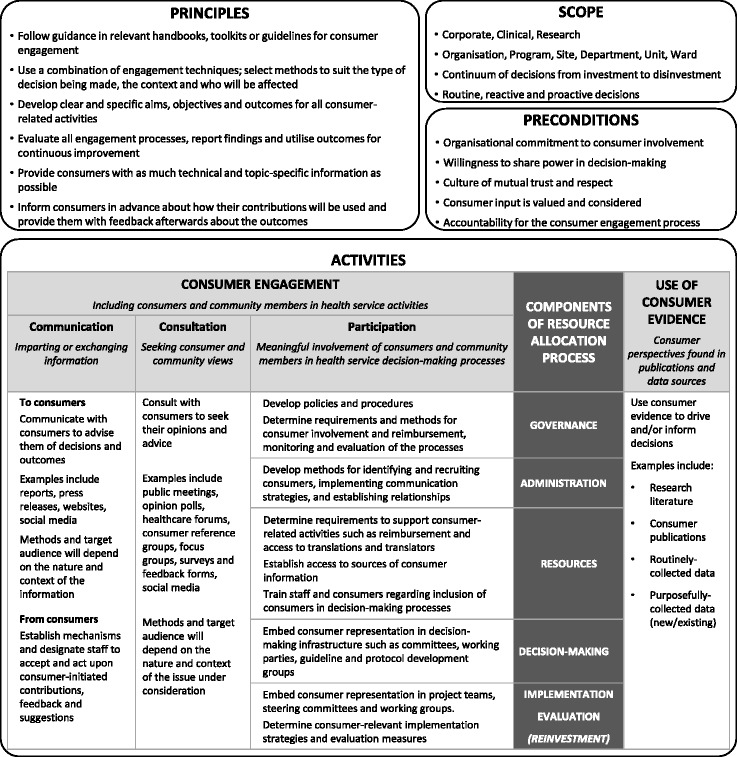
Model for integrating consumer values and preferences into the resource allocation process in a local healthcare setting

The findings are discussed in the context of the proposed model for integrating consumer values and preferences into organisation-wide decision-making for resource allocation (Fig. 5).

Many of the terms related to consumer engagement are reported to be used or interpreted differently and are frequently not defined [25, 31, 60]. The terms used in this model are defined in Table 2.

**Table 2:** Definitions

Health consumers	Patients, potential patients, current and previous users of health services; parents, guardians or carers of patients; organisations representing consumers’ interests; members of the public who are targets of health promotion programs (adapted from ACSQHC [24], CHF [75])
Consumer representatives	Members of a committee, steering group, working party, panel or similar decision-making group who voices the consumer perspective and takes part in the process on behalf of consumers (adapted from CHF [75])
Community	Group of people sharing a common interest including cultural, social, political, health and economic interests and/or a geographic association (adapted from CHF [75])
Consumer engagement	Inclusion of consumers and/or community members in a continuum of activities from passive behaviours such as receiving information, through more active participation, to shared decision-making with equal power. These activities include, but are not limited to, provision of information, consultation, development, participation, collaboration and empowerment (adapted from Sarrami-Foroushani et al [31], O’Mara-Eves et al [33])
Communication	Consumers and/or community members are engaged through imparting or exchanging information. Information can be verbal, written or provided by other methods. Communication can go both ways between consumers and/or community members and health service staff
Consultation	Consumers and/or community members are engaged through requests to provide their views, preferences, comments and suggestions to inform the decision-making process, but the consumers and/or community members may not be engaged in subsequent decision-making or action (adapted from PICS [63], CHF [75])
Participation	Consumers and/or community members are engaged through meaningful involvement in decision-making processes for health policy and planning, healthcare management and service delivery, care and treatment, and the wellbeing of themselves and the community (adapted from Victorian Department of Human Services [28], CHF [75])
Consumer evidence	Consumer opinions, perspectives and preferences found in publications and data sources
Routine decisions	Decisions made on a recurring basis or scheduled via a timetable eg annual budget setting processes, six-monthly practice audits, monthly Therapeutics Committee meetings, reviews of protocols at specified intervals after their introduction, etc [13].
Reactive decisions	Decisions made in response to situations as they arise eg new legislation, product alerts and recalls, applications for new drugs to be included in the formulary, critical incidents, emerging problems, etc [13].
Proactive decisions	Decisions driven by information that was actively sought for this purpose eg accessing newly published synthesised research evidence such as Cochrane reviews to compare against current practice, interrogating routinely-collected datasets to ascertain practices with high costs or high rates of adverse events, etc [13].

*ACSQHC* Australian Council on Safety and Quality in Health Care, *CHF* Consumer Health Forum, *PICS* Paediatric Integrated Cancer Service

### Principles

The list of principles noted below emerged strongly from both the literature and the local interviews and workshop.

There is a plethora of evidence-based handbooks, guidelines and toolkits to assist those seeking to establish a consumer engagement program. Examples are available for international [66, 67], national [69], regional [70], and local levels [71] as well as discipline/condition-specific publications [72].

Evidence suggests that multiple methods should be employed [25, 55, 61, 64] but it is not known which individual strategies are effective [33, 34, 50, 57, 60, 64, 65]. Selecting methods to suit the type of decision being made, the context it is being made in and the people it will affect was proposed.

Activities for consumer engagement need clear and specific aims, objectives and outcomes [50, 54–56]; processes should be evaluated and the outcomes used for continuous improvement [33, 50, 55, 57, 58].

Consumers need to be well-informed for effective engagement [51–53, 55]. It was proposed that they are provided with as much technical and topic-specific information as possible. Consumers should also be informed in advance about how their contributions will be used and provided with feedback afterwards about the outcomes [55].

The importance of training and orientation of consumers for effective involvement is inherent in several of these principles.

### Scope

In the SHARE context, the focus of consumer engagement was in policy and planning, management and service delivery. This is applicable to corporate, clinical and research domains across Monash Health and could be implemented at organisation, program, site, department, unit or ward level.

Disinvestment is undertaken as an independent activity in most examples in the literature. In the SHARE Program, it was thought that disinvestment in isolation from other decision-making processes was artificial and potentially counterproductive and the scope was revised to consider investment and disinvestment within the spectrum of all resource allocation decisions (Fig. 4) [15]. The scope of consumer participation would also reflect this.

Organisational decision-making for resource allocation can be described in three categories: routine, reactive and proactive (Table 2) [13]. Each type of decision provides opportunities for consumer engagement or utilisation of consumer evidence in resource allocation (Fig. 4).

Routine decisions are made on a recurring or scheduled basis and reactive decisions are made in response to situations as they arise. Both would be enhanced by stakeholder’s views. Members of the Consumer Working Group and the Community Advisory Committee agreed that this could be achieved for consumers through engagement of appropriate representatives in the decision-making process or utilisation of published information or locally-collected data.

Proactive decisions arise from information that was specifically ascertained to identify potential for improvement [13]. This information could come from proactive approaches to research, data or stakeholder engagement. Examples provided by respondents are included in Table 3.

**Table 3:** Examples of consumer-related activities generating proactive decisions to drive change

Research	Regularly scanning published research evidence such as reviews by the Cochrane Consumer and Communication Group or publications from relevant consumer agencies for applicability to the local context and comparing the findings with current practice to determine whether there is a need for change
Data	Actively exploring local sources of routinely-collected data such as complaints registers or patient satisfaction surveys for trends or emerging themes that identify opportunities for improvement
Engagement	(Communication) Establishing mechanisms to encourage, accept and act upon consumer-initiated feedback
	(Consultation) Seeking regular consumer feedback to initiate change in targeted areas, for example: ▪ Topics that are important to patients such as pain management and early discharge ▪ Topics that are important to the health service such as cost containment of high volume or high cost procedures where consumer priorities may inform selection of suitable alternatives ▪ Big problems for patients and health services such as falls and medical mishaps ▪ Patients with high usage of health services such as those on renal dialysis ▪ Patients interacting with areas of the health service undergoing frequent or significant change ▪ Patients with cultural, ethnic or language differences that require additional resources

### Preconditions

The same suite of preconditions for effective consumer engagement was identified from the literature and consumer feedback [31, 50–55]. These include organisational commitment to consumer involvement, willingness to share power in decision-making, establishing a culture of mutual trust and respect, placing value on consumer input, and determining accountability for the consumer engagement process.

In addition to these preconditions, the requirement for adequate and appropriate resources to support consumer engagement emerged as a strong message from authors and respondents [33, 50, 57, 58, 68]. This is not included as a precondition in this model as it is addressed as the third component of the resource allocation process.

### Activities

The activities to capture and utilise consumer and community values and preferences are presented in relation to the components of the resource allocation process identified in earlier SHARE work (Fig. 2, Table 1). Only seven of the eight components are included as the purpose of the model is to represent the consumer element of stakeholder engagement as it applies to the other seven components.

Consumer and community perspectives can be identified in two ways: consumer engagement through direct involvement of consumers and community groups and use of consumer evidence through application of previously captured information that reflects consumer and community views and perspectives.

#### Consumer engagement

Engagement covers a range of activities that connect consumers and community members to the health service; from passive behaviours such as receiving information, through more active participation, to shared decision-making with equal power [31, 33, 61]. There are many ways of summarising or classifying engagement strategies. The more detailed classifications include a wide range of activities from exclusion and tokenism through to citizen control [24, 61, 67, 73]. The classification best suited to the Monash Health aim of integrating meaningful consumer engagement into existing systems and processes is based on three categories: communication, consultation and participation [33, 63, 66].

##### Communication

Communication involves imparting or exchanging information which can be verbal, written or provided by other methods. Examples include advertisements, reports, press releases, websites and social media. There are potential opportunities to communicate with consumers and community members within each component of the resource allocation process to advise them of decisions and outcomes. The appropriateness and need for communication will depend on the context and issues under consideration. The target audience for dissemination of information will also be context-specific; for example earlier work with the Monash Health Technology/Clinical Practice Committee determined that decisions about introduction of new health technologies and clinical practices and subsequent reporting on their performance would be sent in annual reports to the Community Advisory Committee [74].

The focus in the literature is on methods for health services to communicate with consumers and community members. Monash Health respondents observed that communication can also be initiated by consumers and community members who wish to communicate with the health service. They noted that for this to be successful, mechanisms to receive consumer-initiated contributions and designated staff to accept and act upon them would be required.

##### Consultation

Consultation is a process of seeking consumer and community views, preferences, comments and suggestions on specified topics to inform the decision-making process [63, 75]. Examples include public meetings, public opinion polls, public hearings, healthcare forums, consumer reference groups, focus groups, surveys, feedback forms and social media. Like communication, there will be opportunities to seek feedback in all of the components of the resource allocation process, dependent on the nature and scope of the decisions being made.

The Monash Health Community Advisory Committee was available for consultation and a database of individuals who had expressed interest in being consumer representatives was also accessible. The other main approach used at Monash Health was to consult with relevant peak bodies or advocacy groups such as Arthritis Victoria or the Australian Association for the Welfare of Child Health.

##### Participation

Participation is meaningful involvement of consumers in health service decision-making processes [28]. Examples include citizen juries, patient panels, consensus conferences, deliberative polls, citizens' dialogues, committee membership and social media. As for communication and consultation, opportunities for participation can arise in the structure and practice elements of each of the components (Table 1) [13]. Examples of potential activities for consumer participation identified by respondents are included in Fig. 5. The importance of providing sufficient resources for training of consumers and staff in effective integration of consumers in decision-making is highlighted.

Consumers were not generally involved in these activities at Monash Health [13]. Although many staff respondents supported consumer participation in decision-making and were planning to act upon this in the future, others thought that consumer representation on their committees would be inappropriate or that consumers had insufficient technical knowledge to participate [13]. There were some notable exceptions: the Human Research Ethics Committee and the Technology/Clinical Practice Committee both had active consumer participation [74] and consumers were integrally involved in all projects undertaken by CCE [45].

#### Use of consumer evidence

Consumer representatives can present the views of consumers through engagement in a range of settings; however they are not the only source of this information. Respondents pointed out that consumer perspectives can also be found in a range of publications and data sources. Consumer evidence could be relevant in all components of the resource allocation process. Proactive use of consumer evidence to initiate and inform resource allocation processes was not evident in the literature.

##### Publications

Many research articles contain qualitative and quantitative information that captures the views of patients, other health service consumers or study subjects. Consumers and community groups also publish discussion papers and opinion pieces in health journals, newsletters of consumer bodies, consumer magazines and similar publications. Some examples of these are included in Table 4.

**Table 4:** Examples of publications with consumer information

Consumer health journals
*'Health Voices – Journal of the Consumers Health Forum of Australia'* is published two times a year to promote debate on health care issues affecting all Australians and of interest to health consumers, government and industry. https://www.chf.org.au/health-voices.php
*'The Australian Health Consumer'* was the official journal of the Consumers Health Forum of Australia from 2001 to 2007. It provided a consumer-focused appraisal of the current and ongoing major health issues of the day in the state, national and international health sector. https://www.chf.org.au/australian-health-consumer.php
*'The Patient: Patient-Centered Outcomes Research'* is the only journal that aims exclusively to examine the needs, values and role of the patient in an increasingly complex healthcare landscape in which funding and decision-making require ever-greater awareness of the patient’s perspective. The journal deals with the full range of patient-centered studies, reviews and commentary ranging through techniques such as conjoint analysis, patient reported outcomes, studies on compliance and satisfaction through to patient-directed health plans and patient literacy. http://www.springer.com/adis/journal/40271
*'​Patient Intelligence'* is an international, peer reviewed, open access journal that characterizes and measures the central role of patient behavior and intention in optimizing healthcare management in all areas of disease and complaint types. An improved understanding of patient intelligence coupled with predictive analysis helps an organization contribute more effectively to achieving better outcomes. The journal is characterized by the rapid reporting of reviews, original research, methodologies, analytics, modeling, clinical studies and patient surveys across all disease areas. Specific topics covered in the journal include: Patient and healthcare literacy, Patient information and healthcare professional communication/interaction, Patient behavior, attitude and trends, Behavior management programs, Quantitative and qualitative research, Data collection systems Business performance management, Benchmarking, assessment and reporting systems, Patient preference, satisfaction, convenience, acceptability and adherence, Patient involvement in the design and development of new treatments and management protocols to optimize outcomes, Decision support systems incorporating patient intelligence. https://www.dovepress.com/patient-intelligence-journal
*'​Patient Preference and Adherence'* is an international, peer reviewed, open access journal that focuses on the growing importance of patient preference and adherence throughout the therapeutic continuum. The journal is characterized by the rapid reporting of reviews, original research, modeling and clinical studies across all therapeutic areas. Patient satisfaction, acceptability, quality of life, compliance, persistence and their role in developing new therapeutic modalities and compounds to optimize clinical outcomes for existing disease states are major areas of interest for the journal. http://www.dovepress.com/aims-and-scope-patient-preference-and-adherence-d16-j20
*'​Patient Related Outcome Measures'* is an international, peer-reviewed, open access journal focusing on treatment outcomes specifically relevant to patients. All aspects of patient care are addressed within the journal and practitioners from all disciplines are invited to submit their work as well as healthcare researchers and patient support groups. Areas covered will include: Quality of life scores, Patient satisfaction audits, Treatment outcomes that focus on the patient, Research into improving patient outcomes, Hypotheses of interventions to improve outcomes, Short communications that illustrate improved outcomes, Case reports or series that show an improved patient experience, Patient journey descriptions or research. http://www.dovepress.com/aims-and-scope-patient-related-outcome-measures-d188-j84
Consumer health organisation newsletters
*'​Consumers Shaping Health'* is a bi-monthly newsletter published by the Consumers Forum of Australia (CHF) for members, consumer representatives and stakeholders in health. It promotes current advocacy work of CHF in its three priority areas: safety and quality in health care; health care for people with chronic conditions; and safe and appropriate use of medicines. https://www.chf.org.au/consumers-shaping-health.php
Cochrane Consumers And Communication Review Group
The Cochrane Consumers and Communication Review Group is part of the international Cochrane Collaboration. The Group coordinates the preparation and publication of systematic reviews of interventions which affect the way people interact with healthcare professionals, services and researchers. These reviews are published in The Cochrane Library. http://cccrg.cochrane.org/welcome
Quality of Care Reports
All Victorian health services are required to publish an annual Quality of Care Report each financial year. The primary audience includes consumers, carers and the health service community. Health services should consult with consumers, carers and community members and/or their Community Advisory Committee about the specific content. Minimum requirements include: ▪ Consumer, carer and community participation ▪ Quality and safety reporting at least four key measures annually (from preventing and controlling healthcare associated infections, medication safety, preventing falls and harm from falls, preventing and managing pressure injuries, clinical indicators for dental services, safe use of blood and blood products) ▪ A review of their local clinical governance policy against the Victorian clinical governance policy framework ▪ A report of the health service’s response to needs of consumers, families or carers and the community across the continuum of care. ▪ Examples or stories that show how these initiatives work in practice.
Other
Consumer driven healthcare is designed to help health care organizations respond effectively to the shift in market power, become consumer-centric, and position themselves to become market leaders in the new consumer-driven market. http://go.galegroup.com.ezproxy.lib.monash.edu.au/ps/i.do?action=interpret&v=2.1&u=monash&it=JIourl&issn=1542-0914&p=AONE&sw=w&authCount=1

##### Data sources

Health facilities routinely collect large amounts of data; within which consumer perspectives are found in incident reports, satisfaction surveys, complaints and compliments. Examples of routinely-collected consumer data noted by respondents are included in Table 5.

**Table 5:** Examples of routinely-collected consumer data

Satisfaction surveys
The Victorian Patient Satisfaction Monitor (VPSM) is a state-wide survey that selects respondents at random; users are sent a unique ID to complete the survey by invitation only.
The Victorian Healthcare Experience Survey (VHES) is a state-wide survey that allows a wide range of people to provide feedback on their experiences and features specialised questionnaires for adult and child inpatients and emergency department attendees, including parents/guardians, and maternity clients. Surveys are distributed in the month following the admission or attendance. People may respond either online or on paper with a freepost return. Surveys are available in English and a range of community languages.
Complaints, Compliments, Comments
Monash Health Complaints, compliments and comments can be made by completing an online form, mailing a printable version of the form, or in person by phone. Complaints are dealt with by the Consumer Liaison Officer on each campus. Details are kept by the Quality Unit.
The Office of the Health Services Commissioner (OHSC) Complainants can also correspond directly with the OHSC. The OHSC’s role is to receive, investigate and resolve complaints from users of health services, to support healthcare services in providing quality healthcare and to assist them in resolving complaints. The legislation also requires that information gained from complaints be used to improve the standards of healthcare and prevent breaches of these standards. This information was the subject of the first study of its kind in Australia in 2014 leading to recommendations for change. The report is available at http://docs.health.vic.gov.au/docs/doc/Study-of-people-lodging-a-complaint-with-the-Victorian-Health-Services-Commissioner
Other
Individual health services and state health departments conduct service reviews, audits and other studies that include patient and consumer information

Monash Health was described as very responsive to incident reports and complaints, reacting quickly and comprehensively, but only on an individual case basis. There were no processes to consider the body of data, seek out patterns or identify areas of concern for further action.

Consumer data is also available from purposefully-collected sources such as surveys, interviews, focus groups and workshops conducted to answer specific questions. This can either be new data collected to address consumer issues under consideration or existing data from previously conducted projects relevant to the current situation. To facilitate use of existing data within the organisation, mechanisms to generate awareness of and enable access to the findings of internal projects would be required. Potential sources of new or existing data identified outside the organisation included self-help groups, peak bodies, health insurance groups, consumer affairs departments and consumer associations. Patient portals and online communities are a combination of in-house and external information where health service consumers can provide information via their own electronic medical record or discussion forums [76].

Monash Health had no systematic approaches to use of locally-collected consumer information or access of external sources.

### Characteristics of the model

The model for consumer engagement is primarily descriptive to enable application in a local healthcare service and allow replication and testing. It was developed using both deductive and inductive methods. Although not based on a specific theory, it has potential to facilitate future theory development and/or testing. Specific characteristics of the model and potential for its use, as discussed in the sections above, are summarised in Table 6 using domains and criteria developed to assess the robustness and utility of proposed models and frameworks [48]. This overview enables potential users to identify whether the model will meet their aims and be applicable to their situation.

**Table 6:** Features of a model for consumer engagement in organisation-wide decision-making for resource allocation in a local health service

Domain	SHARE features
Purpose ▪ descriptive, explanatory or predictive	The model is primarily descriptive to enable replication and testing. There are also some explanatory elements addressed in the relationships between components, for example all elements sit within the context of an organisation-wide program; integration of consumer views and preferences is relevant in all of the other components (governance, administration, resources, decision-making, implementation, evaluation and reinvestment).
Development ▪ deductive or inductive ▪ supporting evidence	Methods used in development were both deductive and inductive. Evidence from the research literature and consultation with health service staff, consumers and community members was used.
Theoretical underpinning ▪ explicit or implicit	No specific theory was used to underpin the model.
Conceptual clarity ▪ well-described, coherent language for identification of elements ▪ strengths and weaknesses of theories ▪ potential to stimulate new theoretical developments	The model overlays the three categories of consumer engagement, Communication, Consultation and Participation, onto the components of organisational infrastructure for resource allocation. The relationships between them are captured in the diagram. Details are provided in the text and in tables. No specific theories were used so no comparisons are made. There is potential for new theoretical developments if: ▪ the model is applied for purposes other than resource allocation for TCPs ▪ the model is applied in settings other than local health service networks ▪ the utility and effectiveness of theories and/or interventions for consumer participation in decision-making are investigated in the settings proposed
Level ▪ individual, team, unit, organisation, policy	The model was developed for organisation-wide implementation in a local health service network for resource allocation decisions. This approach could also be used at a higher (regional, state/provincial, national) or lower (single facility, department or unit) level. It is not designed for application in individual clinical decisions.
Situation ▪ hypothetical, real	The model represents actual settings and contexts in health service decision-making and implementation of change. However it could also be used for teaching or capacity building through hypothetical classroom discussions or simulation exercises.
Users ▪ nursing, medical, allied health, policy makers, multidisciplinary	The model can be used by any decision-makers within the health system. While use of the model could be initiated by any group, engagement and involvement of all relevant stakeholders is an underlying principle of application. The model could be used in policy, management, clinical or research contexts.
Function ▪ barrier analysis ▪ intervention development ▪ selection of outcome measures ▪ process evaluation	The main function is to assist establishment of a consumer engagement program by representing a systematic approach to integration of consumer views and perspectives in organisation-wide decision-making infrastructure and identifying opportunities and methods for consumer engagement in resource allocation decisions. A secondary function is to enable replication and testing.
Testable ▪ hypothesis generation ▪ supported by empirical data ▪ suitable for different methodologies	The model describes the components of organisational infrastructure for resource allocation and settings and opportunities for consumer engagement in this context. A range of hypotheses could be developed for each of these elements and the relationships between them which could be tested in a number of ways using various methodologies.

### Limitations

As the findings and proposed model are based on the infrastructure, practices and experiences of one organisation, generalisability to other health services may be limited. However most of the local findings are consistent with the literature. Some countries, states/provinces or regions have more centralised decision-making and will require consumer participation at the macro rather than meso level. Differences in organisational culture, values and leadership are also likely to affect generalisability and resource-poor countries may not have the same systems and processes or the capacity or capability to introduce activities in the proposed model.

The proposal to embed consumer participation within organisational decision-making may also be incompatible with the system of independent Citizen Councils already established in many settings.

There are many other aspects of consumer involvement that were not addressed such as values [67], principles [28], ladders of participation [73], depth of participation and degree of control [61], barriers and enablers to engagement [35], methods of engagement, and implementation and evaluation of consumer engagement programs [24]. As noted earlier there are also more complex classifications [24, 61, 67, 73]. It has been suggested that “*community engagement in public health is more likely to require a ‘fit for purpose’ rather than ‘one size fits all’ approach*” [33]. The approach taken for this project and the resulting model focused on potential settings and activities for consumer engagement in all of the components of the resource allocation process. It has been tailored to the needs of a health service which had little formal inclusion of consumers in decision-making processes for resource allocation.

### Contribution of this study

This study provides three novel contributions to consumer participation in resource allocation at the local health service level. Firstly, a model is proposed which describes potential underpinning principles, scope, preconditions and activities for successful consumer participation in all components of the resource allocation process at the local health service level. Secondly, the concept of consumer evidence is introduced: sources of consumer views and perspectives found in publications and data sources that can be used systematically and proactively to inform health service decisions. Thirdly, the need for mechanisms within health services to receive and act upon consumer-initiated contributions is identified.

### Implications for policy and practice

The framework for resource allocation (Fig. 2) demonstrates that stakeholder engagement should be integrated within the structure and practice of the other seven components. The proposed model (Fig. 5) illustrates how this might be accomplished for consumer engagement in the resource allocation process. It has been noted that this level of *“institutionalisation of citizen engagement”*, embedding public involvement in decision-making processes with sufficient weight to avoid tokenism and commitment to involvement at an institution-wide level, has rarely been achieved [66, 77]. As an organisation, Monash Health had expressed commitment to consumer engagement, yet it was not widely practiced and many reservations were expressed. Institutions where the culture or leadership do not support consumer engagement are likely to face even greater challenges [35].

The potential to use social media in consumer engagement was not addressed in this project. This concept was not introduced by the project team or the respondents. There are significant potential opportunities for consumer engagement via social media in health generally [78], public relations in hospitals [79, 80], participation in health technology assessments [81], and disinvestment [42, 82].

Consideration of the values and principles underpinning consumer involvement, methods of engagement, and implementation and evaluation of consumer activities will be required when establishing a program incorporating this model.

### Implications for research

This model makes a contribution to the lack of frameworks and models noted in the disinvestment literature [3, 4, 7, 9, 10, 38, 42, 83–86]. It presents opportunities and potential activities for consumer engagement in resource allocation decision-making. Future research could include piloting and refinement of the model in this context or extension into other decision-making settings.

“*Consumer engagement in Australian health policy is poorly understood, inconsistently practiced, and under theorised*” [53]. There is a lack of understanding about how consumer contributions and information flow through the decision-making process and how consumer input contributes to decisions, and a lack of evidence about the effectiveness of different engagement activities [33, 34, 50–60, 64]. The SHARE model provides a structure to focus and facilitate development of hypotheses and testing of interventions in these areas.

Several theoretical approaches have been applied in studies involving consumers or community members in resource allocation decisions; these include decision-making theory [87], deliberation theory [9, 32, 39], social constructionist theory [9], resource allocation theory [40], and prioritisation and quality improvement theories [41]. This model provides opportunities and activities that would enable researchers to investigate the utility and effectiveness of different theories for consumer participation in decision-making.

## Conclusion

Although consumer engagement is increasingly becoming a requirement of publicly-funded health services and documented in standards and policies, participation in organisational decision-making is not widespread. The proposed model presents opportunities and potential activities for consumer engagement in the context of resource allocation.

## Additional file

Additional file 1: Methods. (PDF 425 kb)

## Abbreviations

CCE: Centre for Clinical Effectiveness
SHARE: Sustainability in Health care by Allocating Resources Effectively
TCP: Technology or clinical practice
