# Supplementary material for: Sustainability in health care by allocating resources effectively (SHARE) 4: exploring opportunities and methods for consumer engagement in resource allocation in a local healthcare setting
Source: BMC Health Serv Res. 2017 May 5;17:329. doi: 10.1186/s12913-017-2212-5 (PMC5420096; doi:10.1186/s12913-017-2212-5)
Supplement: Additional file 1: — Methods. (PDF 425 kb) [file 12913_2017_2212_MOESM1_ESM.pdf]

The SHARE Program (Sustainability in Health care by Allocating Resources Effectively) 4: Exploring opportunities and methods for consumer engagement in resource allocation in a local healthcare setting

Additional File 1: Methods

Contents

Systematic review protocol 2

Interviews and workshops 5

References 7

Systematic review protocol

Two-stage review process

Stage 1

Step 1: Identify evidence available.

1. Write search.
2. Run search in websites and medical databases.
3. Apply inclusion and exclusion criteria.

Step 2: Critically appraise synthesised research (ie. EBGs, SRs).

1. Start with the most recent guideline or review and apply standard appraisal criteria.
2. If found to be of high quality, cross check to ensure references from all other synthesised research are included and check for consistency of findings.
3. If not high quality, appraise next most recent guideline or review, and repeat process.
4. Check that all desired settings and outcomes are covered. If not, look for these in other available publications and follow same process
5. If there are inconsistent findings across the existing guidelines and reviews, investigate the possibility of synthesis of the information or whether a new systematic review is required.

Step 3: Identify need for additional SRs.

1. Map available evidence to specific decision-making settings (as per Table A1.1).
2. Identify action required (based on algorithm in Table A1.2). Determine if a good quality but out-of-date SR needs updating, or if a new SR needs to be conducted where no good quality SR was found.

Table A1.1. Map of evidence

Decision-making setting	Consumer engagement techniques implemented and/or evaluated	EBG or frameworks	SRs	RCTs	Other comparative studies	Non comparative studies
Health technologies						
Policies and procedures						
Priority-setting						
Safety and quality						
Service improvement or redesign						
Infrastructure						
Other (to be listed)						

Table A1.2. Further action required to answer questions

<table>
<tr><td colspan="5">Is there any synthesised research evidence available (ie. EBG, SRs)?</td></tr>
<tr><td colspan="3">Yes</td><td colspan="2">No</td></tr>
<tr><td colspan="3">Is this good quality research?</td><td colspan="2">Are RCTs or other comparative studies available?</td></tr>
<tr><td colspan="2">Yes</td><td>No</td><td>Yes</td><td>No</td></tr>
<tr><td colspan="2">Is it current (within 2 years)?</td><td rowspan="3">Undertake new SR</td><td rowspan="3">Undertake new SR</td><td rowspan="3">Look at (low level) non-comparative evidence</td></tr>
<tr><td>Yes</td><td>No</td></tr>
<tr><td>No further action</td><td>Update existing SR</td></tr>
</table>

Stage 2

Update existing high quality reviews and/or undertake new SR if required.

Sources

Searches were undertaken in relevant guideline websites, websites of synthesised evidence, websites of peak health consumer organisations, and government health department websites, and in healthcare databases. Checking of reference lists of article and reports, and links on websites, was also done.

1. Websites

A number of generic guideline clearinghouses and websites of guideline developers have been commonly used by CCE. Additional sources of consumer websites were identified from resource links from the websites of peak health consumer organisations, government health department websites, and by asking experienced consumer advocates. The following websites were searched:

**Guidelines websites**	**Links**
National Health and Medical Research Council (NHMRC)	www.nhmrc.gov.au
New Zealand Guideline Group (NZGG)	http://www.health.govt.nz/about-ministry/ministry-health-websites/new-zealand-guidelines-group
National Guideline Clearinghouse (NGC)	www.guidelines.gov
Scottish Intercollegiate Guidelines Network	www.sign.ac.uk
UK National Institute for Health and Clinical Excellence (NICE)	www.nice.org.uk
Guidelines Advisory Committee	www.gacguidelines.ca/index.cfm
The Joanna Briggs Institute	joannabriggs.org/
The Guidelines International Network	www.g-i-n.net
**Databases and search engines**	
TRIP Database	www.tripdatabase.com
Intute Database	www.intute.ac.uk
UK National Health Service (NHS) Evidence	www.evidence.nhs.uk/default.aspx
Google	www.google.com
**Government and consumer health organisation websites**	
Consumers Health Forum of Australia (CHF)	www.chf.org.au
Picker Institute Europe	www.pickereurope.org
Health Issues Centre	www.healthissuescentre.org.au
Victorian Government Department of Health	www.health.vic.gov.au
Australian Government Department of Health and Ageing	www.health.gov.au
Health Canada	www.hc-sc.gc.ca/index-eng.php
UK Department of Health	www.dh.gov.uk

2. Health databases

The following medical databases were searched: All EBM (including Cochrane Database of Systematic Reviews, DARE, CENTRAL, and ACP Journal Club), Medline(R) 1950 to present with daily updates and Medline(R) in-process and other non-indexed citations, CINAHL, and EMBASE.

Search terms

Search string for websites consists of the following combination of a "consumer" term and an "engagement" term. Where website search engines could not support truncation all terms were entered in full.

Consumer terms	***Engagement terms***
Consumer, Consumers	Engagement, Engaging, Engage
Community, Communities	Participation, Participating, Participate
Citizen, Citizens	Involvement, Involving
Patient, Patients	Consultation, Consulting
Public	Deliberating, Deliberation, Deliberate
	Input

For searching the medical databases, an initial scoping exercise identified that using a comprehensive search using keywords and MeSH headings returned a very high number of articles that would be impractical to sift through. MeSH headings were used to restrict the number of retrieved results. “Consumer participation” and “health planning” MeSH headings were used to capture most of the relevant literature. Because the All EBM database does not have MeSH headings, the search terms used for websites as listed previously was used to obtain relevant articles.

Inclusion and exclusion criteria

Inclusion and exclusion criteria were established *a priori* and were applied by a single reviewer.

	Inclusion	Exclusion
**Population**	There are two populations that are targeted:<br>▪ Consumers, the public, patients, or the community<br>▪ Health service staff and groups who are decision-makers at an organisation-wide level or multi-departmental level, particularly managers and senior staff	Health service staff or committees who are making decisions at a single departmental level or lower level.
**Intervention / indicator**	Consumer or community engagement strategies for organisation-wide or multi-departmental decision-making. The documents must provide information on one or both of the following:<br>▪ Implementation methods<br>▪ Evaluation methods and measures used for the consumer engagement strategies for organisation-wide decision-making.	Consumer engagement strategies aimed at health or medical research projects.<br>Consumer engagements strategies aimed at specific disease group services (eg. cancer services).<br>Documents not providing details on implementation or evaluation methods.
**Comparison / control**	Any consumer engagement strategy.<br>No consumer engagement.	
**Outcomes**	How consumers contributed to the decision-making process.<br>Methods of successful implementation of consumer engagement.<br>Methods for evaluating the consumer engagement process.<br>Methods for reporting to consumers how their input contributed to the decision-making process.	
**Setting**	Health service setting.<br>Organisation-wide or multi-departmental decision-making processes, consultations, committees, or working groups. Situations may include clinical safety committees, consumer advisory committees, or health technology and clinical practice committees.	Focused only on specific disciplines (eg. mental health) or for specific projects (eg. research projects).
**Study design**	Evidence in order of preference:<br>1. Evidence-based guidelines<br>2. Systematic reviews<br>3. Randomised controlled trials<br>4. Lower quality comparative studies<br>5. Local and colloquial evidence (eg. case studies)	Narrative reviews<br>Non-evidence-based guidelines<br>Frameworks not based on evidence (theoretical only)<br>Letters and editorials<br>Opinion pieces
**Language**	English	Languages other than English
**Time period**	From 1990 onwards. From advice from experienced consumer advocates and officers, there has only been tangible action in consumer engagement in healthcare within the past 10 years, and up to a maximum of 20 years.	

Data collection and analysis

All quality appraisals were conducted by a single reviewer in consultation with colleagues as required using CCE templates appropriate to the study design. An initial scoping exercise identified a variety of evidence sources for consumer engagement, such as evidence based guidelines, SRs, RCTs, and case studies. For this appraisal, there are a variety of situations where consumer engagement may occur and there may be multiple consumer engagement techniques that may have been used for a specific situation. A two-staged approach was undertaken where synthesised evidence was critically appraised, and gaps in the evidence identified, as stated in Methods 1.

If there are gaps in the evidence, an update of existing high quality reviews can be done and/or a new SR undertaken.

Interviews and workshops

Semi-structured discussions with Consumer Working Group

Aim: To identify potential opportunities and methods for consumer participation and sources of consumer information.

Inclusion criteria: Consumer representatives with experience in organisation-wide decision-making related to resource allocation.

Sampling: Convenience sampling was used. Three consumer representatives who met the inclusion criteria were known to the project team (as members of committees overseeing introduction of new TCPs and development of policies and procedures), two of them were on the SHARE Steering Committee.

Approach: The three representatives were approached personally and invited to participate.

Design: Workshop 1 addressed the question: How can we capture consumer perspectives and include in decisions related to organisation-wide systems and processes? Prompts for discussion included

Methods of involvement: Who? How? Use of research literature and local/other data: What? Where? How? Who else to talk to? and Things we haven't thought of? Workshop 2 considered and refined the findings of Workshop 1 and added further detail.

Data collection: Group discussions were held at meetings convened for this purpose. Project team members took notes.

Respondent validation: Drafts were sent to the interviewees for clarification, comment and/or amendment as required.

Analysis: Responses were collated and added to findings from the other sources which were then analysed thematically by content analysis.

Response rate: All members of the Consumer Working Group participated in both workshops.

Representativeness of sample: The consumer representatives were experienced in health service decision-making and familiar with organisational systems and processes.

Semi-structured interviews with staff responsible for consumer-related activities

Aim: To identify consumer-related activities within the organisation

Inclusion criteria: Staff with responsibility for consumer-related activities

Approach: Invitations for interview were sent to the Monash Health Quality Manager and Consumer Engagement Manager.

Interview schedule: What consumer-related activities occur within the organisation? What are your thoughts on findings from Consumer Working Group and interviews with Monash Health staff? Who else to talk to? Things we haven't thought of?

Data collection: Individual interviews were held at meetings convened for this purpose in the interviewee's office. One member of the project team was both interviewer and note taker.

Respondent validation: Drafts were sent to the interviewees for clarification, comment and/or amendment as required.

Analysis: Responses were collated and added to findings from the other sources which were then analysed thematically by content analysis. Responses were summarised by emergent themes and presented in detailed reports used for project decision-making and planning.

Response rate: Both invitees participated.

Representativeness of sample: The interviewees were the senior managers responsible for consumer participation in the organisation.

Structured workshop with Community Advisory Committee

Aims: To identify current consumer engagement activities, barriers and enablers to effective participation in these situations and the needs of consumers in order to contribute effectively; to identify sources of consumer information and data and how these sources can be used to drive decision-making; and to seek feedback on a draft model for consumer engagement in generic health service decision-making

Inclusion criteria: The Community Advisory Committee is a legislated advisory body to the health service Board providing consumer, carer and community perspectives. This group provides a consultation service to health service staff engaging in consumer-related activities.

Approach: A request for consultation was completed. A workshop was held at a meeting convened for this purpose.

Design: The project team delivered a presentation that included the background and aims of SHARE, potential decision-making settings identified in earlier SHARE work, and findings from the literature review, interviews and consultation with staff and consumers regarding current and potential consumer participation in decision-making at Monash Health. This was followed by a structured discussion on the following topics:

- Committees and Working Parties: What would consumers need to contribute effectively? What are the barriers and enablers to effective participation? Other thoughts?
- Consultation: Are there particular areas we should focus on? What would consumers need to contribute effectively? What are the barriers and enablers to effective participation? Other thoughts?
- Using our consumer data: Sources we have identified. Are there others? Should there be others? How can we use this information to drive decision-making? How should consumers be involved in this process?
- Using our other data: Sources we have identified. How can we use this information to drive decision-making? How should consumers be involved in this process?
- Consumer literature: Suggestions. How can we use this information to drive decision-making? How should consumers be involved in this process?
- Is there anything else we have missed?

Data collection: Project staff took notes.

Analysis: Responses were collated and added to findings from the other sources which were then analysed thematically by content analysis.

Response rate: 6 of the 14 committee members attended the workshop

Structured interviews with staff authorised to make decisions on behalf of the organisation
**Aim:** These interviews were conducted to address several research questions. The aim related to the research question in this paper was to ascertain current practice in consumer involvement in organisational decision-making and implementation and evaluation of change.<br>**Inclusion criteria:** Staff and consumers authorised to make decisions regarding resource allocation for health technologies and clinical practices at organisation-wide level in group or individual settings.<br>**Sampling:** Purposive and snowball sampling was used.<br>▪ Twenty-two committees were initially identified from a governance structure diagram. A further 20 were identified through a snowballing method by asking participants in the subsequent interview process, senior managers and Quality Unit staff if they were aware of others. Fourteen of the 42 potential committees met the inclusion criteria (Capital Expenditure, Falls Prevention, Information Systems Governance, Joint Program Quality and Safety, Medication Safety, Operating Suite Product Evaluation, Nurse Standardisation of Practice, Resuscitation, Skin Integrity and Pressure Ulcer, Sterilising Services, Technology and Clinical Practice, Therapeutics and Transfusion Committees and the Executive Management Team).<br>▪ Approved Purchasing Units (APUs) have delegated authority from the Board to commit the organisation to a legal and/or financial obligation such as issuing a purchase order or signing a contract. Of the nine APUs, two had been included in the group decision-making committees (Capital Expenditure Committee and Executive Management Team) and five others met the inclusion criteria (Pharmacy, Health Technology Services, Equipment Services, Procurement and Clinical Purchasing, and Materials Management).<br>▪ Clinical managers from one clinical program selected for its high use of health technologies were identified from the program's intranet page. Individuals were selected purposively to represent all levels within the program's decision-making hierarchy; medical and surgical sub-specialties, nursing and quality management; and a range of campuses.<br>**Approach:** Personalised email invitations from the project team were sent to the Chair, Executive Sponsor and/or Secretary of 14 committees, managers of 5 APUs and 9 managers from the selected clinical program. Approval from the Nursing and Medical Program Directors was sought before approaching individuals from the selected program.<br>**Interview schedule:** Questions were based on a theoretical framework [1]. They were piloted with one committee and refined before being used in subsequent interviews. Consumer-related questions were a subset of the broader interviews. The full interview schedule is available [1].<br>**Data collection:** Interviews were approximately 1 hour long and were conducted in the interviewee's office or suitable meeting room. Interviews were not taped or transcribed but detailed notes were taken. Two CCE staff members attended, one as interviewer and one as note taker.<br>**Respondent validation:** Drafts were sent to the interviewees for clarification, comment and/or amendment as required.<br>**Analysis:** Final interview notes were collated and organised in MS Word and Excel using the elements of the theoretical framework. Concepts related to consumer engagement for resource allocation in the local healthcare context and relationships between these concepts were extracted and collated with findings from the other sources and developed into a framework<br>**Response rate:** Representatives of 13 of the 14 committees, all 5 APU managers and 9 clinical managers participated. One committee Chair did not respond to the invitation for interview; due to lack of time no representative of this committee was interviewed. A surgical sub-specialty department head was unable to attend their interview and was replaced by a medical sub-specialty department head who was available at short notice.<br>**Representativeness of sample:** Almost all eligible committees and all eligible APUs were represented. The clinical managers represented Program Directors, Department Heads, Unit/Ward Managers and ancillary services; medical (n=4), nursing (n=4) and quality management (n=1) staff; in a range of sub-specialties across multiple campuses.
**Structured interviews with staff members with experience in disinvestment projects**
**Aim:** These interviews were conducted to address several research questions. The aim related to the research question in this paper was to learn about consumer involvement in previous disinvestment projects at Monash Health.<br>**Inclusion criteria:** Staff who had undertaken projects to remove, reduce or restrict current practices (the term 'disinvestment' was not used in Monash Health projects).<br>**Sampling:** Purposive and snowball sampling was used. Relevant projects were initially identified by members of the SHARE Steering Committee and interviewees in the committee review process noted above. A snowballing method was employed by asking participating project representatives if they knew of any other relevant projects. Nineteen potential projects were identified, 13 met the inclusion criteria.<br>**Approach:** Personalised email invitations from the project team were sent to project managers of 13 relevant projects. Project managers or Department/Unit Heads were sought as key contacts; however a representative of the project team was accepted when a senior staff member was unavailable.<br>**Interview schedule:** Questions were designed to explore project governance, use of routinely-collected hospital data, other local data and research evidence in the development and implementation of projects; barriers and enablers to successful project implementation; what staff would do again and what they would do differently. The full interview schedule is available [1].<br>**Data collection:** Interviews were approximately 1 hour long and were conducted in the interviewee's office or suitable meeting room. Interviews were not taped or transcribed but detailed notes were taken. Two CCE staff members attended, one as interviewer and one as note taker.<br>**Respondent validation:** Drafts were sent to the interviewees for clarification, comment and/or amendment as required.<br>**Analysis:** Final interview notes were collated and organised in MS Word and Excel using the elements of the theoretical framework. Concepts related to consumer engagement for resource allocation in the local healthcare context and relationships between these concepts were extracted and collated with findings from the other sources and developed into a framework<br>**Response rate:** Representatives of 10 projects participated based on interviewee's and interviewer's availability<br>**Representativeness of sample:** The process was designed to be illustrative and did not seek to comprehensively identify all projects. A number of project topics across a range of clinical areas were included.

References

1. Harris C, Allen K, Waller C, Brooke V. Sustainability in Health care by Allocating Resources Effectively (SHARE) 3: Examining how resource allocation decisions are made, implemented and evaluated in a local healthcare setting BMC health services research. 2017;(Details TBA).
